# Brain Tumor Segmentation Using Deep Capsule Network and Latent-Dynamic Conditional Random Fields

**DOI:** 10.3390/jimaging8070190

**Published:** 2022-07-08

**Authors:** Mahmoud Elmezain, Amena Mahmoud, Diana T. Mosa, Wael Said

**Affiliations:** 1 Computer Science Department, Faculty of Science, Tanta University, Tanta 31527, Egypt; 2 College of Computer Science and Engineering, Taibah University, Yanbu 966144, Saudi Arabia; wael.mohamed@zu.edu.eg; 3 Faculty of Computers and Information, KafrElSheikh University, Kafr El-Sheikh 32626, Egypt; amena_mahmoud@fci.kfs.edu.eg (A.M.); diana.mosa@yahoo.com (D.T.M.); 4 Computer Science Department, Faculty of Computers and Informatics, Zagazig University, Zagazig 44511, Egypt

**Keywords:** medical image segmentation, brain tumor, deep capsule network, latent-dynamic condition random field

## Abstract

Because of the large variabilities in brain tumors, automating segmentation remains a difficult task. We propose an automated method to segment brain tumors by integrating the deep capsule network (CapsNet) and the latent-dynamic condition random field (LDCRF). The method consists of three main processes to segment the brain tumor—pre-processing, segmentation, and post-processing. In pre-processing, the N4ITK process involves correcting each MR image’s bias field before normalizing the intensity. After that, image patches are used to train CapsNet during the segmentation process. Then, with the CapsNet parameters determined, we employ image slices from an axial view to learn the LDCRF-CapsNet. Finally, we use a simple thresholding method to correct the labels of some pixels and remove small 3D-connected regions from the segmentation outcomes. On the BRATS 2015 and BRATS 2021 datasets, we trained and evaluated our method and discovered that it outperforms and can compete with state-of-the-art methods in comparable conditions.

## 1. Introduction

Brain tumor occurrences are substantial contributors to the worldwide mortality rate. Brain tumors kill more individuals under the age of 40 in Australia than any other malignancy, according to the Cure Brain Cancer Foundation. Furthermore, despite the amazing improvement in the survival rate of other forms of cancer in Australia, its survival rates are poor and have not improved substantially in the last 30 years [1]. The World Health Organization (WHO) divides brain tumors into four categories based on cell origin and behavior, ranging from the least aggressive to the most aggressive.

Tumors classified as grades I or II (low grade (LG) tumors) are non-malignant brain tumors, whereas malignant brain tumors are classified as grades III or IV (high grade (HG) tumors) [2]. HG tumors are life-threatening and have a maximum life expectancy of two years, but LG tumors have a significantly longer life expectancy of up to ten years.

Segmentation of brain tumors using neuro imaging modalities is a significant step in improving disease diagnosis, treatment planning, monitoring, and clinical trials. To determine the location and size of a brain tumor, precise segmentation is required. Brain tumors, on the other hand, have characteristics that make perfect segmentation challenging [3]. Tumors can appear in a variety of locations and can be of virtually any shape or size. Furthermore, they are typically poorly contrasted, and the intensity value of a tumor may overlap with the value of healthy brain tissue.

**Figure 1:**
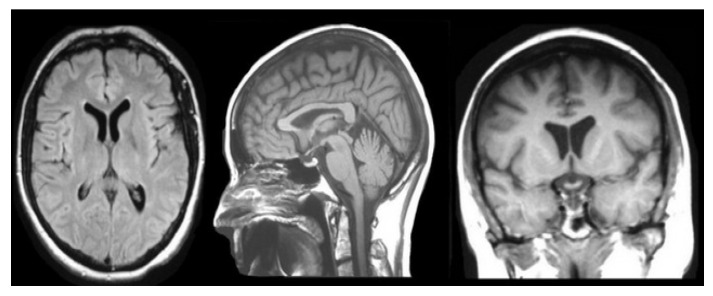
The brain, spinal cord, and vascular anatomy are all thoroughly detailed [4].

Magnetic resonance imaging (MRI) is one of the most frequently performed diagnostic procedures in neurology and neurosurgery [4]. The advantage of magnetic resonance imaging (MRI) is that it can observe the anatomy in all three planes: axial, sagittal, and coronal. It also provides good details about the architectures of the brain, spinal cord, and vascular system (Figure 1).

Neuroimaging techniques to segment brain tumors are important steps toward improving disease diagnosis, treatment planning, monitoring, and clinical trial participation. To determine the location and size of a brain tumor, it is necessary to conduct precise segmentation. While brain tumors have unique characteristics that make precise segmentation difficult, they are not frequent. The development of tumors can occur in a variety of locations and can take on practically any shape or size. Furthermore, they are frequently poorly contrasted, and the intensity levels of malignancies and healthy brain tissues may be similar in magnitude.

However, manual contouring of brain tumors has been attempted in the past, but it is time-consuming and has low reliability across a wide range of operators [5,6]. Numerously-automated or semi-automated approaches were developed in recent years (see, for example, reviews in [7]), but challenges, such as the lack of accuracy when compared to a specialist’s evaluation, have prevented their widespread clinical applications among neuro-diologists and neurosurgeons. In a therapeutic setting, a fully automated procedure that provides accurate and meaningful segmentation findings is necessary. The proposed method makes use of multimodal brain tumor image segmentation (BRATS) dataset [8] published by the University of Pennsylvania Center for Biomedical Image Computer and Analytics, and headed “Description of the present suggested model”. Our research contributes significantly by proposing an automated brain tumor segmentation method that integrates deep CapsNet with LDCRF. The method is trained and tested on the BRATS 2015 and BRATS 2021 datasets, where it outperforms state-of-the-art algorithms in similar settings and has the potential to be competitive. The recommended method is explained in Section 3 of our study. Our experimental results are presented in Section 4. The suggested method is concluded in Section 5.

## 2. Related Work

Among the datasets published by the University of Pennsylvania Center for Biomedical Image Computer and Analytics (which is further described in the dataset section) is the benchmark brain tumor segmentation (BraTS) dataset, which hosts an annual contest to find the best models on the data. Table 1 and Table 2 show summaries of dataset specifications. Researchers have been able to develop and test cutting-edge brain tumor segmentation algorithms using the BraTS dataset, which has grown in size over time (2012–2021), as the number of patient cases has increased and the data split has improved, allowing them to create and test cutting-edge brain tumor segmentation algorithms.

These algorithms can be divided into two groups:

- Generative models that classify brain voxels based on image attributes and that require prior knowledge via probabilistic atlas image registration and take into account the spatial distributions of tissues and their appearances.
- Discriminative models that define brain voxels based on image features and learn characteristics from manually annotated data are used to classify and learn characteristics from manually annotated data.

Table 1 introduces a summary of the current surveys.

Throughout the testing, validation, and training sets, as well as the targeted tasks of the BraTS instance, Table 2 shows the distribution of BraTS datasets over time and throughout all three sets since the start of the initiative in 2012, and continuing through 2020.

The training and testing datasets for BraTS first two versions (2012–2013) were 35 mp MRI patient scans and 15 mp MRI patient scans, respectively, for the first two iterations. It was in [17] that the findings and conclusions of the two initial editions were presented, and it has since become the most public and downloaded manuscript in the IEEE *TMI* journal, demonstrating the scientific research community’s interest in BraTS effort as a community benchmark and publicly available database.

The following three BraTS instances (2014–2016) saw significant growth in the datase, as well as the addition of longitudinal mp MRI scans in two successive waves. A contribution to the first wave of growth came from the Cancer Imaging Archive (TCIA) repository, followed by a contribution from Heidelberg University, and a contribution to the second wave of growth came from the University of CBICA (Pennsylvania’s Center of Biomedical Image Computing and Analytics, Chicago, IL, USA), which was established in 2016 [18,19,20]. As a result of the review of the BraTS (2012–2013) findings, BraTS (2014–2016) incorporates the ground truth data generated using the label fusion of the best-performing techniques, which were previously unavailable.

As part of the BraTS dataset validation set, the University of Alabama in Birmingham (UAB) and CBICA@UPenn submitted a dataset in 2017, following the machine learning paradigm of training, validation, and testing datasets. This helped to fine-tune the algorithm by following the machine learning paradigm of training, validation, and testing datasets. As compared to the previous year, the number of reported cases more than doubled, reaching 477 in 2017. Because of contributions from institutions, such as the MD Anderson Cancer Center in Texas, the Tata Memorial Center in India, and the Washington University School of Medicine in St. Louis, among others, the number of instances increased to 542 in 2018 [21].

Regarding tumor segmentation, a U-Net model design [22] is one of the most effective neural network model architectures currently available. To begin with, CNN layers are contracted to collect context (essential attributes, such as glioma area borders), and then CNN layers are expanded to localize predictions over the entire image (21 scans).

Feng et al. explored the use of ensemble techniques developed across numerous U-Net models in 2020, and discovered that the combined forecasts outperformed the predictions of single U-Net models [23]. As a result, because the group’s sub-models are trained to suit both enhancing and non-enhancing tumors at the same time, each sub-model is unable to segment a specific region of the glioma, which is a significant limitation.

The CNN-transformer combined model, termed BiTr-Unet [24], was proposed by Qiran Jia and Hai Shu to perform brain tumor segmentation with respect to multi-modal MRI data, developed in collaboration with Google. On the BraTS 2021 validation dataset, the proposed BiTr-Unet achieves excellent performance, with mean Dice scores (0.9076, 0.8392, 0.8231), as well as mean Hausdor distances (4.5322, 13.4592, 14.9963) to the whole tumor, tumor core, and enhancing tumor, respectively.

Antonio et al., using magnetic resonance imaging (MRI), suggested an accurate brain tumor segmentation method that has a wide range of applications [25], including radiosurgery planning. Automatic segmentation has been made possible by breakthroughs in artificial intelligence, notably deep learning (DL), which have paved the way for the abolition of labor-intensive and operator-dependent manual segmentation. It was the DL model using pre-gadolinium and post-gadolinium contrast T1 and T2 FLAIR sequences that the others outperformed, earning the highest Dice scores of 0.878 for the total tumor, 0.732 for the tumor core, and 0.699 for the active tumor. There was minimal difference in performance when T1 or T2 sequences were not present, while FLAIR and T1C were important considerations.

## 3. Suggested Method

Models trained on feature extraction were used to segment the entire tumor regions from input MR images. These trained models were also put to the test against the extracted features of testing MR images. Every trained model produced an output that represented the entire tumor region. Each model was trained using 80% of the data and then tested using the remaining 20%.

**Figure 2:**
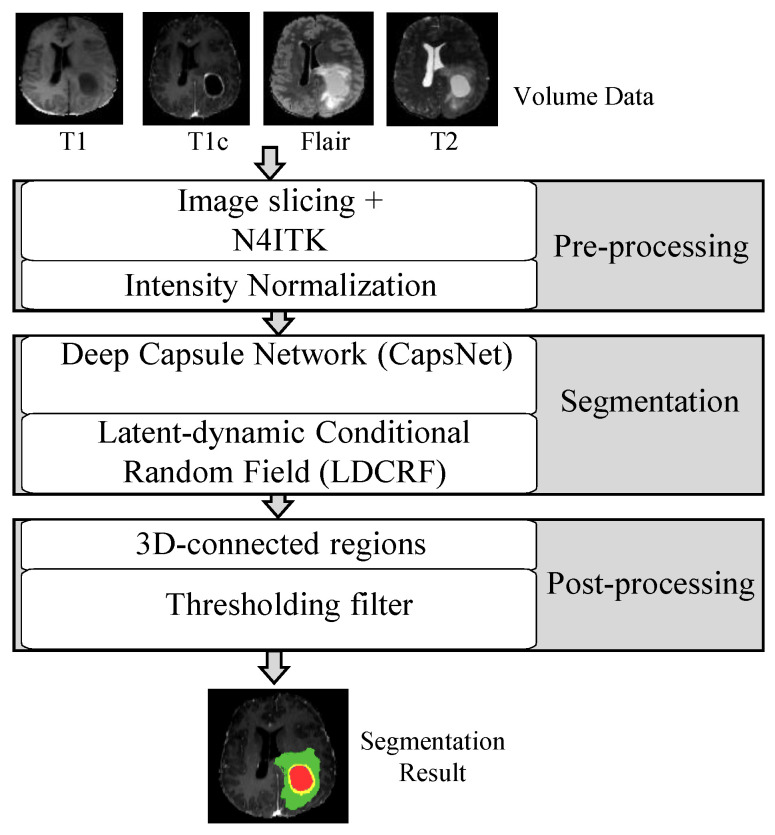
Structure of our suggestion with respect to three key steps.

Tumor segmentation entails assigning a class to every pixel in the image or 3D volume–voxel. In the MRI image, a binary semantic segmentation for the brain tumor is performed using deep learning algorithms. This binary segmentation labels each pixel as a tumor or background. Pre-processing, segmentation, and post-processing stages are key processes of our proposed method, which integrated CapsNet and LDCRF. We will go over each step in detail in the sections below (Figure 2).

### 3.1. Pre-Processing

The memory amount that is required to process and store 3D volumes is one of the difficulties in medical image segmentation. The problem is solved by using the overlap-tile strategy to stitch test patches together to form a segmented test volume after training the network on image patches. It also avoids border artifacts in the neural network by using the valid part of the convolution [26]. According to inhomogeneity throughout the tumor scans, caused via different magnetic field movements, dealing with MRI data is the second most difficult task. This means that absolute intensity values in various MR images, or even in the same MR images, have no meaning for the tissue they come from because they do not stay the same. It is important to perform proper pre-processing on MR images. There are many biases in the MRI scans of patients that make obtaining accurate segmentation results difficult. The intensity values of the same tissues change a lot from time to time due to bias field distortion. We first employed N4ITK to alleviate the bias field of every MR image in this study [27,28].

**Figure 3:**
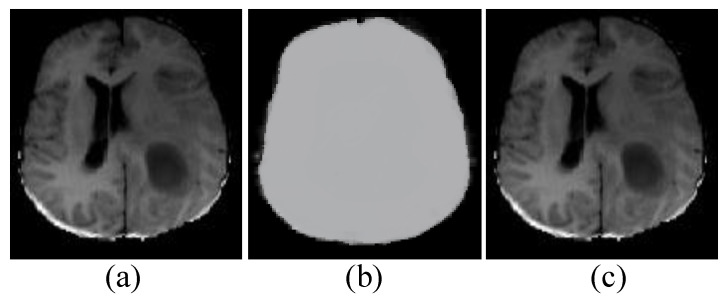
The impact of MR image intensity bias and the outcome of bias field correction. (**a**) before (**b**) the estimated bias field and (**c**) after the N4ITK bias field correction.

The N4 bias field correction algorithm is a widely used approach for correcting low-frequency intensity non-uniformity, often known as a bias or gain field, in MRI image data. This approach does not require tissue classification and assumes a simple parametric model. This filter requires one input image that is impacted by a bias field that we want to fix. To achieve pixel-to-pixel compatibility, the mask picture and the primary input image must occupy the same physical space. The revised deviation is then divided by subtracting the gray-value of the highest frequency and then normalizing the intensity. The revised deviation is denoted by ξ, and the MR image ready for normalization is denoted by *V*, which is made up of voxels $v_{1},v_{2},\ldots ,v_{N}$. Each voxel’s intensity value $v_{k}$ is given as $I_{k}$. The revised deviation ξ can then be determined using the following equation:

(1) $$\xi =\sqrt{\sum_{k=1}^{N}{\left(I_{k}-\hat{I}\right)}^{2}/N}$$

where $\hat{I}$ signifies the highest frequency’s gray value. We also modified the intensity range of the MR pictures to 0–255 linearly in order to analyze them as common images. In most circumstances, the highest frequency’s gray value is near the strength of white matter. As a result, converting the gray value of the greatest frequency to the same level is analogous to converting white matter intensities to the same level. Similar intensities in multiple MR images can have the same tissue significance after normalizing the revised deviation (Figure 3).

### 3.2. Segmentation

For the purpose of segmenting gliomas from MRI images, we used a deep capsule network in conjunction with the latent conditional random field in this study. The network was trained using T1, T1 post-contrast, Flair, and T2 sequences using 2D axial slices (240 × 240). The network’s architecture allows for semantic segmentation, which involves classifying all voxels in a slice. As a result, Deep CapsNet-based networks have faster inference times than typical patch-based CNNs.

**Figure 4:**
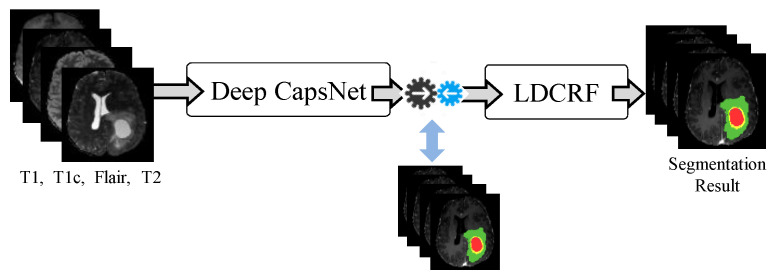
Our brain tumor segmentation model’s structure.

As depicted in Figure 4, the segmentation step of our model consists of two parts: a Deep CapsNet and a latent-dynamic conditional random field. Image patches for Deep CapsNet and slices for LDCRF were used to learn the proposed model. CapsNet was first learned using image patches. Then, with the settings of the CapsNet fixed, we used image slices from the axial view to learn LDCRF-CapsNet. Finally, the image slices were applied to the entire network to fine-tune it. The brain images were segmented slice by slice during the testing phase. The axial view was used to extract all of the slices. Following that, we went over each component of the suggested segmentation model in detail.

#### 3.2.1. Deep Capsule Network

**Figure 5:**
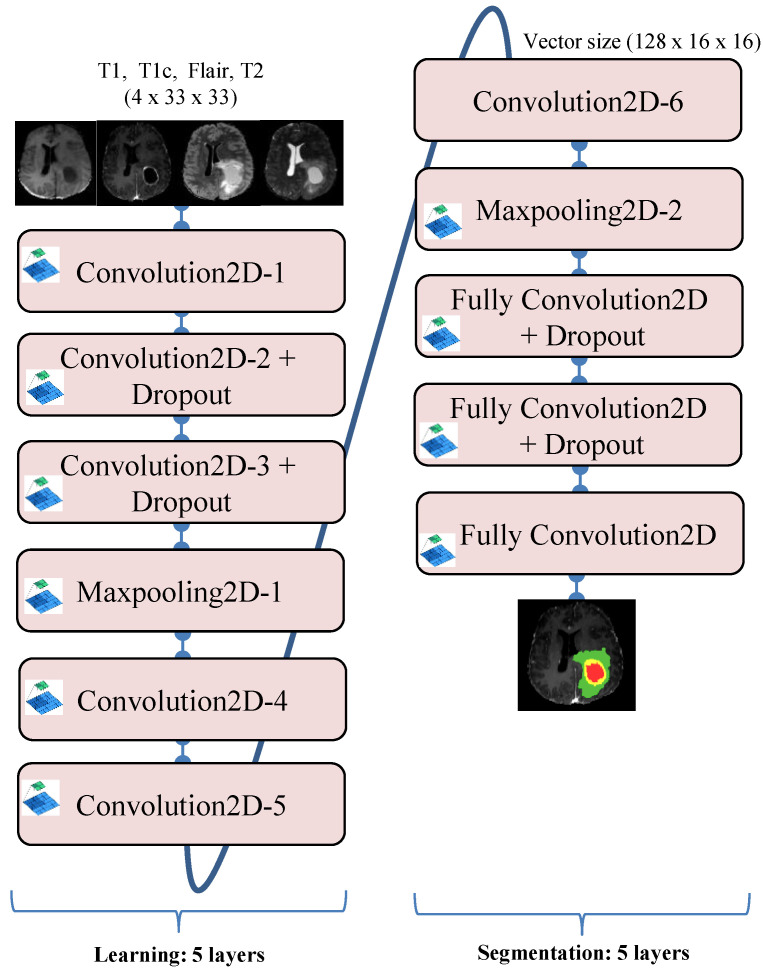
Structureof transfer learning and the segmentation CNN model.

In our suggested method, a patch size 33 × 33 is chosen from a 240 × 240 image size. The patch extraction lowers the complexity of a convolution neural network by reducing the number of parameters. As a result, the model requires a specific set of high-level parameters for tumor segmentation. The category of a particular voxel is proportional to the category of its neighbors. Patches are sometimes utilized for training purposes. We utilized a deep CapsNet with an 11-layer architecture that encapsulated the transfer learning and segmentation layers. The transfer learning CNN model included three convolution layers at first, then a max-pooling layer and two convolution layers. The output of the transfer learning method was passed to the segmentation process, consisting of one convolution layer, followed by one max-pooling layer, and then three completely convolution layers in the form of a vector of 128 × 16 × 16. In the convolution and max-pooling layers, a tiny kernel of 3 × 3 was employed as the filter, and in two completely convolution layers, a dropout of rate 0.5 was used (Figure 5).

Overfitting is a significant stumbling block to excellent performance in medical image segmentation utilizing a convolutional neural network. To alleviate the problem of overfitting, a dropout layer was implemented. A dropout layer with a rate of 0.8 was used in the transfer learning layer. The segmentation layer had a dropout rate of 0.5 for another dropout layer. The dropout rate had a significant impact on the complexity of segmentation layers and the layers of a retrained convolutional neural network (CNN) that used transfer learning.

#### 3.2.2. LDCRF

**Figure 6:**
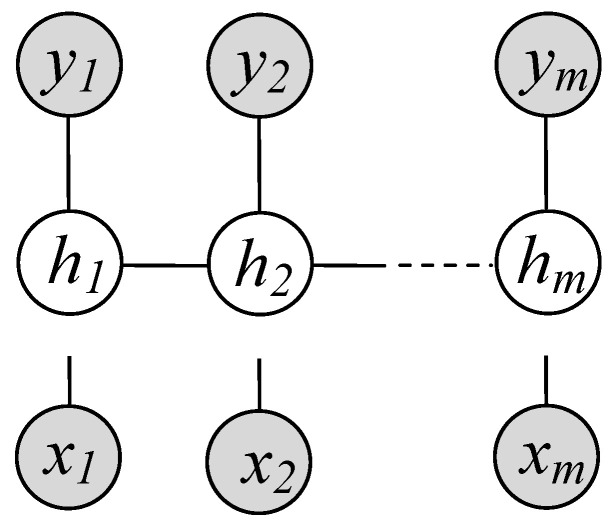
LDCRF model, in which $x_{j}$ is to the value of jth the corresponding observation, the hidden state of $x_{j}$ is to $h_{j}$. $y_{j}$ represents a label of $x_{j}$, such that the gray circle is to the observation data.

Tumors in MR brain imaging are classified and assigned to different classes. LDCRF is used as a classifier to assign each pixel to its label at this stage. LDCRF is a set of undirected graphical models to label the sequential data [29,30]. Each label is associated with a single color pixel (Figure 6). Because they provide a class label for each observation, LDCRF model is naturally utilized for classifying unsegmented sequences. Furthermore, throughout the training and testing processes, it efficiently models infer-tumor action sequences.

We suppose that the sub-structure hidden variables denote vectors *h* = $h_{1},h_{2},\ldots ,h_{m}$ for each sequence. Given the observation sequence *x*, the probability p(y|x,θ) is determined as follows:

(2) $$p\left(y|x,\theta \right)=\sum_{h:\forall h_{i}\in H_{yi}}p\left(h|x,\theta \right)$$

here, $h_{i}$ is one of a set of possible hidden states with respect to the label $y_{j}$.

(3) $$p\left(h|x,\theta \right)=\frac{1}{Z(x,\theta )}\exp\left(\sum_{i=1}^{n}F_{\theta }\left(h_{i-1},h_{i},x,i\right)\right)$$

where the number of transition feature functions $\theta =(\lambda_{1},\lambda_{2},\ldots ,\lambda_{N_{f}};\mu_{1},\mu_{2},\ldots ,\mu_{N_{g}})$ is assigned by $N_{f}$. The number of label feature function is to $N_{g}$, and the length of an observation length of sequence *x* is *n*. Here, the $F_{\theta }$ is estimated as;

(4) $$\begin{array}{c} F_{\theta }\left(h_{i-1},h_{i},x,i\right)=\;\;\;\;\;\;\\ \sum_{f}\lambda_{f}t_{f}\left(h_{i-1},h_{i},x,i\right)+\sum_{g}\mu_{g}s_{g}\left(h_{i},x,i\right)\;\; \end{array}$$

where $t_{f}\left(h_{i-1},h_{i},x,i\right)$ is to a function of transition features at position *i* and position i−1, and $s_{g}\left(h_{i},x,i\right)$ denotes a function of the state feature in location *i*, $\lambda_{f}$, and $\mu_{g}$ denote the functions of the transition and label features, respectively. The normalized factor, Z(x,θ), can be determined in the following way:

(5) $$Z\left(x,\theta \right)=\sum_{h}\exp\left(\sum_{i=1}^{n}F_{\theta }\left(h_{i-1},h_{i},x,i\right)\right)$$

To learn the LDCRF model, the parameter $\theta =(\lambda_{1},\lambda_{2},\ldots ,\lambda_{N_{f}};\mu_{1},\mu_{2},\ldots ,\mu_{N_{g}})$ is calculated using the learning data $D={\left\{\left(x^{\left(j\right)},y^{\left(j\right)}\right)\right\}}_{j=1}^{T_{d}}$. Here, $x^{\left(j\right)}$ is a training set observation sequence (i.e., intensity color for each pixel), $y^{\left(j\right)}$ refers to the matching label sequence of an observation sequence $x^{\left(i\right)}$. Moreover, $T_{d}$ represents the number of learning sequences. Equation (6) calculates the objective function of the learning parameter θ, which maximizes the log-likelihood of the learning dataset.

(6) $$\begin{array}{cc} L\left(\theta \right) & =\sum_{j=1}^{T_{d}}\log\;p\left(y^{\left(j\right)}|x^{\left(j\right)},\theta \right)\\ & =\sum_{j=1}^{T_{d}}\left(\sum_{i=1}^{n}F_{\theta }\left(h_{i-1}^{\left(j\right)},h_{i}^{\left(j\right)},x^{\left(j\right)},i\right)-\log Z\left(x^{\left(j\right)},\theta \right)\right) \end{array}$$

The BFGS optimization algorithm, which requires 300 iterations to converge, can be used to accomplish the likelihood maximization [31].

(7) $$\begin{array}{c} \frac{\partial L(\theta )}{\partial \theta }=\sum_{j=1}^{T_{d}}(\sum_{i=1}^{n}\frac{\partial F_{\theta }\left(h_{i-1}^{\left(j\right)},h_{i}^{\left(j\right)},x^{\left(j\right)},i\right)}{\partial \theta }\;\;-\\ \sum_{x}p\left(h|x^{\left(j\right)}\right)\sum_{i=1}^{n}\frac{\partial F_{\theta }\left(h_{i-1},h_{i},x^{\left(j\right)},i\right)}{\partial \theta }) \end{array}$$

Regarding the inference LDCRF model, a set of matrices was constructed with respect to the probability of the label sequence *y*, given an observation sequence *x* [31]. Special start $h_{0}$ and end $h_{n+1}$ states have been introduced to various expressions to make them easier to understand. These are fictitious states. Assume that Equation (3) gives us p(h|x,θ). $M_{i}\left(x\right)$ is at the |S×S| matrix, defined as follows to every position *i* in the observation sequences;

(8) $$M_{i}\left(h^{\prime },h|x\right)=\exp\left(F_{\theta }\left(h^{\prime },h,x,i\right)\right)$$

Such that $S=h_{1},h_{2},\ldots ,h_{l}$ denotes a set of training data labels. The number of labels is represented by *l*. The labels of *S* at time *i* are $h^{\prime }$ and *h*. The conditional probability p(y|x,θ) can be calculated using this notation as follows:

(9) $$p\left(y|x,\theta \right)=\frac{\prod_{i}^{n+1}M_{i}\left(h_{i-1},h_{i}|x\right)}{Z(x,\theta )}$$

The inputs of products to these matrices represent the normalization Z(x,θ):

(10) $$Z\left(x,\theta \right)={\left(\prod_{i=1}^{n+1}M_{i}\left(i\right)\right)}_{start,stop}$$

#### 3.2.3. Merging of Deep CapsNet and LDCRF

The proposed brain tumor segmentation network is built using Deep CapsNet and LDCRF, with CapsNet giving the prior probability of assigning every label to every voxel. The prior prediction findings are regarded as the inverse of the single term LDCRF. Furthermore, LDCRF can optimize the segmentation results worldwide based on the position and the intensity information displayed in the pre-processed MR image for each voxel. The spatial consistency and appearance of the segmentation results can then be assured.

### 3.3. Post-Processing

We fixed the labels of some pixels after post-processing the segmentation results by deleting tiny 3D-connected regions and removing small 3D-connected areas using a simple threshold. A small portion of the learning dataset was used to validate the values of these thresholds.

## 4. Results and Discussions

The brain tumor image segmentation (BRATS) benchmark is an image segmentation benchmark for brain tumors. On this benchmark, connected with the international conference of Medical Image Computing and Computer-Assisted Intervention (MICCAI), the majority of segmentation techniques related to the state-of-the-art has been tested. Our investigations are based on the RENA-ASNR-BRATS MICCAI’s 2015 and BRATS 2021 datasets. They include four MRI modalities (T1, T1c, flair, and T2), and the segmentation labels of learning data. The data were randomly separated into learning and testing sets, with learning data accounting for 80% of all images dataset. The testing dataset was carried out of 20% of the data and was used to verify our suggested approach. Only the training data had pixel labels, which were separated into four categories: necrosis, edema, enhancing, and non-enhancing tumors. A complete tumor (i.e., all classes of tumor), core tumor (enhancing, non-enhancing, and necrosis), and enhancing tumor were the three classes used to evaluate the suggested method. Our system was implemented in the Matab2021a, hp core-i7 computer with 16 GB of RAM that included a variety of ways and pre-trained models for implementing the deep neural network.

### 4.1. Evaluation: BRATS 2015 Dataset

**Figure 7:**
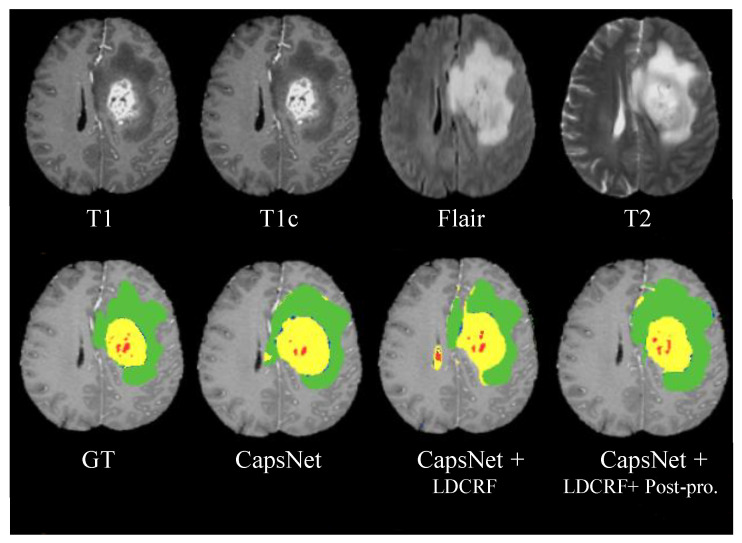
Results of the segmentation on MR images with respect to MICCAI BRATS 2015 dataset. Four input modalities are shown in the top row from left to right (T1, T1c, Flair, T2). Ground truth (GT) as well as segmentation outcomes utilizing CapsNet, CapsNet + LDCRF, and CapsNet + LDCRF + post-processing, respectively, in the bottom row (left to right) with red label 1 (necrosis), green label 2 (edema), blue label 3 (non-enhancing), and yellow label 4 (enhancing).

The dataset of BRATS 2015 contains 274 learning MR images, where 220 are to HGG and 54 to LGG [32]. The modalities available for every patient include T1 weighted, gadolinium (post-contrast T1-weighted), T2-Flair, and T2-weighted (Figure 7). The segmentation results were assessed using three criteria: specificity, sensitivity, and dice similarity coefficient (DSC) are factors to consider [32]. Each metric was computed with respect to three different types of tumor areas: complete, core, and enhancing tumors. The entire tumor region is made up of necrosis, edema, and enhancing and non-enhancing cores. The core area contains enhancing, non-enhancing cores, and necrosis. The enhancing core refers to the only part of the enhancing region. By overlapping the expected output image *O* with the manual segmented label *L*, the dice score was calculated. The dice similarity coefficient was calculated by intersecting two images, pixel-by-pixel, estimated by;

(11) $$DSC=2\cdot \frac{\left|O\cap L\right|}{\left|O|+|L\right|}$$

The sensitivity of the accurately-classified tumor labeling is a measure of the correctness. It indicates how effectively the model is at detecting the tumor in a particular image; it is expressed as:

(12) $$Sensitivity=\frac{\left|O\cap L\right|}{\left|L\right|}$$

Specificity is correctly used to calculate the accuracy of the recognized labels to the typical output class. It assesses how well the model is at finding the tumor in its predetermined location. It is calculated using the intersection of the expected normal label $O_{*}$ and the actual normal label $L_{*}$.

(13) $$Specificity=\frac{|O_{*}\cap L_{*}|}{|L_{*}|}$$

Figure 7 depicts the suggested method’s segmentation results, emphasizing the efficacy of the CapsNet, CapsNet + LDCRF, and CapsNet + LDCRF + post-processing models. The four MRI modalities employed are shown in the top row, while the ground truth and segmentation findings are shown in the bottom row.

According to the BRATS 2015 Challenge dataset, Table 3 displays Dice scores, sensitivity, and specificity of CapsNet, CapsNet + LDCRF, and CapsNet + LDCRF + Post-processing. Table 3 shows that LDCRF can significantly enhance the accuracy of segmentation and post-processing can further enhance segmentation accuracy. On all three areas of both datasets, CapsNet + LDCRF + post-processing performs best. The results of our investigation of the BRATS 2015 dataset are shown in Table 4 and were compared against state-of-the-art methodologies reported to the BRATS challenge benchmark. According to the tumor core and enhancement areas, the proposed methods outperform state-of-the-art approaches, while producing equivalent outcomes to the complete tumor area. The enhanced performance to the core and enhancing classes, as well as the high specificity values, are the highlights of this effort. As indicated in Table 4, the majority of the outcomes reflect the DICE score [32] utilized for comparison.

Kuan-Lun Tseng et al. [33] introduced a deep convolutional neural network that focuses on global information rather than patch-based information. It generated the best outcomes for the overall tumor areas. L.L. Folgoc et al. [7] suggested a cascade of lifted decision forests (CLDF) as an enhanced implementation for the decision forests. E.A.R. Piedra [34] described a technique of variability characterization of tumor boundaries (VCTB) based on the varied nature of brain imaging. Bi Song [35] used the anatomy-guided (AG) approach to locate the region of interest (ROI) and then carried out the random forests to determine the final segmentation outcome. Adria Casamitjana introduced 3-D CNN (3DNet3), which carried out the best segmentation outcomes on the database of BRATS 2015 [36]. Adria Casamitjana suggested three potential 3D structures; however, only the best was utilized for comparison with our study. A deep convolutional neural network (DCNN)-based automated brain tumor segmentation algorithm was introduced by [32], in which a patch-based strategy combined with an inception process was employed to learn the deep network via collecting two co-centric patches for varied sizes from the input image. Batch normalization, drop-out, the inception module, and non-linear activation are all modules in deep neural networks that have been integrated to create a novel ILinear nexus design. The results reveal that the majority of models and approaches focus on the total tumor Dice score while enhancing the tumor area has the lowest outcomes. In the core and augmenting regions, the proposed methodology, on the other side, outperforms state-of-the-art procedures while delivering comparable outcomes over the whole tumor region. In general, the suggested method uses a CPU to segment the entire brain in 4–6 min, which is significantly faster than the method by S. Hussain et al. [32] (with an average running time of 8 min).

### 4.2. Evaluation: BRATS 2021 Dataset

**Figure 8:**
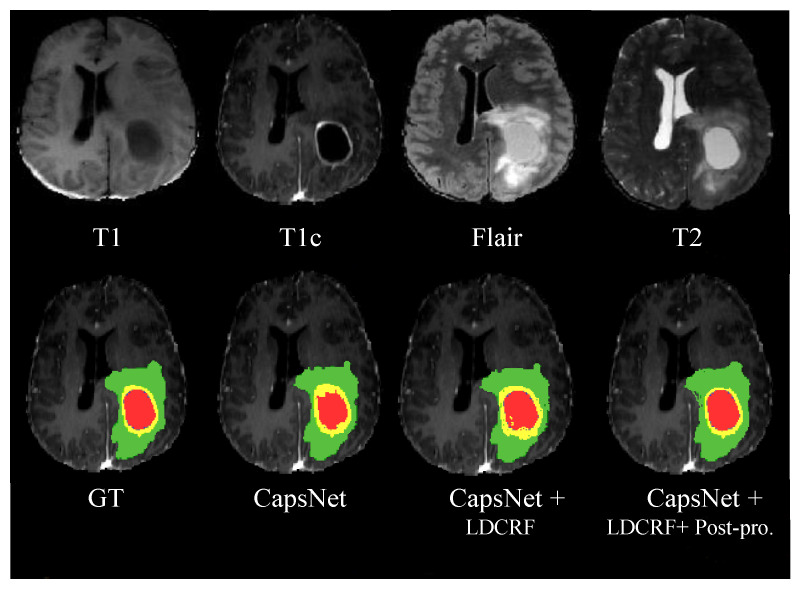
Results of segmentation on MR images with respect to the MICCAI BRATS 2021 dataset. Four input modalities are shown in the top row from left to right (T1, T1c, Flair, T2). Ground truth (GT) as well as segmentation outcomes utilizing CapsNet, CapsNet + LDCRF, and CapsNet + LDCRF + post-processing, respectively, in the bottom row (left to right) with red label 1 (necrosis), green label 2 (edema), blue label 3 (non-enhancing), and yellow label 4 (enhancing).

In [37], the BRATS 2021 dataset contains pre-operative MR scans for 2000 participants separated into testing, validation, and training cohorts. The ground truth labels for training segmentation models are given to 1251 subjects for the segmentation task. Each subject is stripped to four 3D MRI modalities (T1, T1c, FLAIR, and T2). 240 × 240 × 155 pixels are the dimensions of the input image. The ground truth labels are available to 585 subjects for the classification task. Because the testing cohort is not accessible, the classification task’s validation data contain image data of 87 people, which are given with no ground truth labels but can be utilized for evaluating the performance of the model [38]. Figure 8 shows the segmentation results of the proposed architectures, highlighting the effectiveness of models CapsNet, CapsNet + LDCRF, and CapsNet + LDCRF + post-processing. The four MRI modalities employed are shown in the top row, while the ground truth, in addition to the segmentation outcomes for the proposed methods, are shown in the bottom row.

The Dice scores of CapsNet, CapsNet + LDCRF, and CapsNet + LDCRF + post-processing on the BRATS 2021 Challenge dataset are shown in Table 5. As can be observed, LDCRF significantly improves segmentation accuracy, and post-processing improves segmentation accuracy even more.

In all three areas of both datasets, CapsNet + LDCRF + post-processing perform best. The suggested methods outperform state-of-the-art models for core and enhancing tumor areas and produce comparable outcomes through tumor areas. Along with the high specificity values, this work highlights the performances of core and enhancing classes.

The results of the comparisons with other approaches are described in Table 6, where the Dice score is reported in the majority of the cases. According to the segmented brain tumor on multi-modal MRI data, a CNN-transformer hybrid model called BiTr-Unet is provided [24]. BiTr-Unet is a reliable and robust network for extracting both long-range and local dependencies for 3D MRI scans, according to the validation data. In [39], a method for 3D brain tumor segmentation from multimodal was used, as well as participation in the BraTS 2021 challenge. They used an encoder-decoder-based segmentation network but changed the training process to reduce redundancy during perturbations. In [40], the joint graph convolution–image convolution neural network on the Brain Tumor Segmentation (BraTS) 2021 challenge is considered. Each brain is modeled as a graph made up of separate picture areas, first segmented using a graph neural network (GNN). Following that, a voxel (simple) convolutional neural network (CNN) refines the tumorous volume identified by the GNN, resulting in the final segmentation. Furthermore, due to the coarse supervoxel generation step, this approach has difficulty in precisely delineating specific tumors and tumor compartment boundaries. The presented method, on the other hand, outperforms current procedures in the core and enhancing areas, whereas it yields comparable outcomes through the complete tumor area.

## 5. Conclusions

Segmenting brain tumors is considered a challenging research project in medical image processing because of the unpredictable and fluctuating sizes and shapes of tumors. In the proposed research, an automated method to segment the brain tumor is presented to address this challenge, by merging the deep capsule network (CapsNet) and latent-dynamic condition random field (LDCRF) data sets (LDCRF). There were three main steps in our method—pre-processing, segmentation, and post-processing—to carry out the segmentation. Before normalizing the intensity of each MR image, N4ITK employed pre-processing to make the bias field of every image accurate. To perform the segmentation operation, CapsNet was trained using image patches. After that, we employed image slices from the axial view to learn the LDCRF-CapsNet to train the integrating method while keeping the settings of the CapsNet constant. We next post-processed the segmented outcomes by eliminating small 3D-connected areas and applying a simple threshold method to fix the labels of the pixels that were selected for processing. Our methodology was trained and tested on the datasets from BRATS2015 and BRATS 2021, which are both publicly available. When compared to previous studies, the findings are encouraging. The proposed method can be improved in the future by integrating additional features and employing a variety of classifiers and optimization techniques, among other things.

## Figures and Tables

**Table 1:** The current surveys.

The Title of the Survey	Publish Year	Remarking
State-of-the-art survey on MRI brain tumor segmentation [8]	2013	Prior to 2013, a list of segmentation methods was developed.
A survey of MRI-based brain tumor segmentation methods [2]	2014	A survey about segmentation approaches of brain MRI, which was carried out in the years preceding 2014; the results were published in 2014.
Recent advances in convolutional neural networks [9]	2015	An investigation was conducted into the application of convolutional neural networks in language processing, computer vision, and speech recognition.
Probabilistic machine learning and artificial intelligence [10]	2015	An overview of stochastic machine learning techniques and the applications of these approaches.
A survey on deep learning in medical image analysis [11]	2017	A detailed assessment of deep learning algorithms for medical image processing is presented, including both theoretical and practical considerations.
Deep learning for brain MRI segmentation: state-of-the-art and future directions [12]	2018	This work provides deep learning algorithms for brain MRI segmentation that relies on machine learning.
Deep convolutional neural networks for the brain image analysis on magnetic resonance imaging: a review [13]	2018	Convolutional neural networks (CNNs) are discussed in relation to their usage in evaluating magnetic resonance imaging (MRI) of the brain.
Deep learning for generic object detection: a survey [14]	2018	A detailed survey of deep learning-based object detection methodologies.
A guide to deep learning in healthcare [15]	2019	Deep learning techniques to boost healthcare applications are discussed in this survey.
Deep learning-based brain tumor Segmentation: a survey [16]	2020	A comprehensive evaluation of deep learning to segment the brain tumor.

**Table 2:** BraTS dataset specification from 2012 to 2020.

Year	Total Data	Training Data	Validation Data	Testing Data	Processes	Data Type
2012	50	35	N/A	15	Segmentation	Pre-operative only
2013	60	35	N/A	25	Segmentation	Pre-operative only
2014	238	200	N/A	38	Disease progression and segmentation	Longitudinal
2015	253	200	N/A	53	Disease progression and segmentation	Longitudinal
2016	391	200	N/A	191	Disease progression and segmentation	Longitudinal
2017	477	285	46	146	Segmentation and survival prediction	Pre-operative only
2018	542	285	66	191	Segmentation and survival prediction	Pre-operative only
2019	584	300	80	204	Segmentation and survival prediction	Pre-operative only
2020	620	340	80	200	Segmentation and survival prediction	Pre-operative only

**Table 3:** On the MICCAI BRATS 2015 dataset, segmentation outcomes with respect to the Dice score, sensitivity, and specificity.

Methods	Dice			Sensitivity			Specificity		
	Complete	Core	Enhancing	Complete	Core	Enhancing	Complete	Core	Enhancing
CapsNet	0.82	0.80	0.81	0.79	0.76	0.78	0.86	0.84	0.82
CapsNet + LDCRF	0.87	0.84	0.82	0.85	0.82	0.80	0.88	0.86	0.83
CapsNet + LDCRF + Post-processing	0.91	0.86	0.85	0.88	0.84	0.83	0.93	0.90	0.86

**Table 4:** On the BRATS 2015 Challenge dataset, comparison with different approaches.

Methods	Dice		
	Complete	Core	Enhancing
Kuan-Lun Tseng et al. [33] (DCNN)	0.85	0.68	0.87
L. L. Folgoc et al. [7] (CLDF)	0.79	0.67	0.70
A. R. P. Piedra et al. [34] (VCTB)	0.74	0.54	0.54
Bi Song et al. [35] (AG)	0.85	0.70	0.73
C. Adria et al. [36] (3DNeT3)	0.92	0.84	0.77
S. Hussain et al. [32] (ILinear)	0.86	0.87	0.90
Our Method	0.91	0.86	0.85

**Table 5:** In the MICCAI BRATS 2021 dataset, segmentation outcomes with respect to the Dice score, sensitivity, and specificity.

Methods	Dice			Sensitivity			Specificity		
	Complete	Core	Enhancing	Complete	Core	Enhancing	Complete	Core	Enhancing
CapsNet	0.83	0.81	0.80	0.80	0.77	0.78	0.87	0.85	0.82
CapsNet + LDCRF	0.87	0.85	0.83	0.85	0.83	0.81	0.88	0.86	0.84
CapsNet + LDCRF + Post-procesing	0.92	0.88	0.85	0.90	0.86	0.85	0.93	0.91	0.87

**Table 6:** On the BRATS 2021 Challenge dataset; comparison with different approaches.

Methods	Dice		
	Complete	Core	Enhancing
Q. Jia and H. Shu [24] (BiTr-Unet)	0.91	0.84	0.82
M. M. R. Siddiquee and A. Myronenko [39] (SegResNet)	0.93	0.89	0.86
C. Saueressig et al. [40] (GNN-CNN)	0.89	0.81	0.73
Our Method	0.92	0.88	0.85

## Data Availability

All data have been present in main text.

## References

[1] Perry A., Reifenberger G., von Deimling A., Figarella D., Cavenee W.K., Ohgaki H., Wiestler O.D., Kleihues P., Ellison D.W. (2016). The 2016 World Health Organization Classification of Tumors of the Central Nervous System: A summary. Acta Neuropathol..

[2] Liu J., Li M., Wang J., Wu F., Liu T., Pan Y. (2014). A Survey of MRI-Based Brain Tumor Segmentation Methods. Tsinghua Sci. Technol..

[3] Menze B.H., Leemput K.V., Lashkari D. Segmenting Glioma in Multi-Modal Images using a Generative Model for Brain Lesion Segmentation. Proceedings of the MICCAI-BRATS.

[4] Yushkevich P.A., Pashchinskiy A., Oguz I., Mohan S., Schmitt J.E., Stein J.M., Zukić D., Vicory J., McCormick M., Yushkevich N. (2019). User-Guided Segmentation of Multi-modality Medical Imaging Datasets with ITK-SNAP. Neuroinformatics.

[5] Awad N., Mahmoud A. (2021). Improving the Quality of Reconstructed Image by Using Hybrid Compression Based on DWT-DCT Techniques. Comput. Mater. Cont..

[6] Bakas S., Akbari H., Sotiras A., Bilello M., Rozycki M., Kirby J.S., Freymann J.B., Farahani K., Davatzikos C. (2017). Advancing The Cancer Genome Atlas glioma MRI collections with expert segmentation labels and radiomic features. Sci. Data.

[7] Folgoc L.L., Nori A.V., Alvarez-Valle J., Lowe R., Criminisi A. Segmentation of brain tumors via cascades of lifted decision forests. Proceedings of the MICCAI-BRATS Workshop.

[8] Gordillo N., Montseny E., Sobrevilla P. (2013). State of the art survey on mri brain tumor segmentation. Magn. Reson. Imag..

[9] Gu J., Wang Z., Kuen J., Ma L., Shahroudy A., Shuai B., Liu T., Wang X., Wang G., Chen T. (2015). Recent advances in convolutional neural networks. Pattern Recogn..

[10] Ghahramani Z. (2015). Probabilistic machine learning and artificial intelligence. Nature.

[11] Litjens G., Kooi T., Bejnordi B.E., Adiyoso A.A.S., Ciompi F., Ghafoorian M., van der Laak J.A.W., van Ginneken B., Sanchez C.I. (2017). A survey on deep learning in medical image analysis. Med. Image Anal..

[12] Akkus Z., Galimzianova A., Hoogi A., Rubin D.L., Erickson B.J. (2017). Deep learning for brain mri segmentation: State of the art and future directions. J. Digit. Imag..

[13] Bernal J., Kushibar K., Asfaw D.S., Valverde S., Oliver A., Marti R., Llado X. (2018). Deep convolutional neural networks for brain image analysis on magnetic resonance imaging: A review. Artif. Intell. Med..

[14] Liu L., Ouyang W., Wang X., Fieguth P., Chen J., Liu X., Pietikainen M. (2018). Deep learning for generic object detection: A survey. arXiv.

[15] Esteva A., Robicquet A., Ramsundar B., Kuleshov V., DePristo M., Cui C., Chou K., Corrado G., Thrun S., Dean J. (2019). A guide to deep learning in healthcare. Nat. Med..

[16] Wang Z.L., Kuen J., Ma L., Shahroudy A., Shuai B., Liu T. (2020). Deep Learning Based Brain Tumor Segmentation: A Survey. arXiv.

[17] Menze B.H., Jakab A., Bauer S., Kalpathy J., Farahani K., Kirby J., Burren Y., Porz N., Slorboom J., Wiest R. (2015). The Multimodal Brain Tumor Image Segmentation Benchmark (BRATS). IEEE Trans. Med. Imag..

[18] Urban G., Bendszus M., Hamprecht F., Kleesiek J. Multimodal Brain Tumor Segmentation using Deep Convolutional Neural Networks. Proceedings of the MICCAI-BRATS.

[19] Dvorak P., Menze B. Structured prediction with convolutional neural networks for multimodal brain tumor segmentation. Proceedings of the MICCAI-BRATS.

[20] Kamnitsas K., Ferrante E., Parisot S., Ledig C., Nori A.V., Criminisi A., Rueckert D., Glocker B. (2016). DeepMedic on Brain Tumor Segmentation. Proceedings of the Brainlesion: Glioma, Multiple Sclerosis, Stroke and Traumatic Brain Injuries.

[21] Daimary D., Bora M.B., Amitab K., Kandar D. (2020). Brain Tumor Segmentation from Multi Modal MR images using Fully Convolutional Neural Network. Procedia Comput. Sci..

[22] Li X., Chen H., Qi X., Dou Q., Fu C.W., Heng P.A. (2018). H-DenseUNet: Hybrid Densely Connected UNet for Liver and Tumor Segmentation From CT Volumes. IEEE Trans. Med. Imaging.

[23] Feng X., Tustison N.J., Patel S.H., Meyer C.H. (2020). Brain Tumor Segmentation Using an Ensemble of 3D U-Nets and Overall Survival Prediction Using Radiomic Features. Front. Comput. Neurosci..

[24] Jia Q., Shu H. (2021). BiTr-Unet: A CNN-Transformer Combined Network for MRI Brain Tumor Segmentation. arXiv.

[25] Ieva A.D., Russo C., Jian A., Bai M.Y., Magnussen J.S., Quian Y. (2021). Application of deep learning for automatic segmentation of brain tumors on magnetic resonance imaging: A heuristic approach in the clinical scenario. Neuroradiology.

[26] Ronneberger O., Fischer P., Brox T. U-Net: Convolutional Networks for Biomedical Image Segmentation. Proceedings of the International Conference on Medical Image Computing and Computer-Assisted Intervention.

[27] Shen G., Ding Y., Lan T., Chen H., Qin Z. Brain Tumor Segmentation Using Concurrent Fully Convolutional Networks and Conditional Random Fields. Proceedings of the 3rd International Conference on Multimedia and Image Processing (ICMIP 2018).

[28] Sille R., Choudhury T., Chauhan P., Sharma D. (2021). A Systematic Approach for Deep Learning Based Brain Tumor Segmentation. Ing. Syst. Inform..

[29] Elmezain M. (2017). Invariant color features-based foreground segmentation for human-computer interaction. Math. Methods Appl. Sci..

[30] Elmezain M., Al-Hamadi A. (2018). Vision-Based Human Activity Recognition Using LDCRFs. Int. Arab J. Inform. Technol..

[31] Elmezain M., Ibrahem H.M. (2020). Retrieving Semantic Image Using Shape Descriptors and Latent-Dynamic Conditional Random Fields. Comput. J..

[32] Hussain S., Anwar S.M., Majid M. (2018). Segmentation of glioma tumors in brain using deep convolutional neural network. Neurocomputing.

[33] Tseng K.L., Lin Y.L., Hsu W., Huang C.Y. Joint Sequence Learning and Cross-Modality Convolution for 3D Biomedical Segmentation. Proceedings of the IEEE Conference on Computer Vision and Pattern Recognition (CVPR).

[34] Piedra E.A.R., Ellingson B.M., Taira R.K., El-Saden S., Bui A.A.T., Hsu W. (2016). Brain Tumor Segmentation by Variability Characterization of Tumor Boundaries. Proceedings of the Brainlesion: Glioma, Multiple Sclerosis, Stroke and Traumatic Brain Injuries.

[35] Song B., Chou C.R., Chen X., Huang A., Liu M.C. (2016). Anatomy-Guided Brain Tumor Segmentation and Classification. Proceedings of the Brainlesion: Glioma, Multiple Sclerosis, Stroke and Traumatic Brain Injuries.

[36] Casamitjana A., Puch S., Aduriz A., Vilaplana V. (2016). 3D Convolutional Neural Networks for Brain Tumor Segmentation: A Comparison of Multi-resolution Architectures. Proceedings of the Brainlesion: Glioma, Multiple Sclerosis, Stroke and Traumatic Brain Injuries.

[37] Baid U., Ghodasara S., Bilello M., Mohan S., Calabrese E., Colak E., Farahani K., Kalpathy-Cramer J., Kitamura F.C., Pati S. (2021). The rsna-asnr-miccai brats 2021 benchmark on brain tumor segmentation and radiogenomic classification. arXiv.

[38] RSNA-MICCAI Brain Tumor Radiogenomic Classification Challange. https://www.kaggle.com/c/rsna-miccai-brain-tumor-radiogenomic-classification/.

[39] Siddiquee M.M.R., Myronenko A. (2021). Redundancy Reduction in Semantic Segmentation of 3D Brain Tumor MRIs. arXiv.

[40] Saueressig C., Berkley A., Munbodh R., Singh R. (2021). A Joint Graph and Image Convolution Network for Automatic Brain Tumor Segmentation. arXiv.

